# Improved gene co-expression network quality through expression dataset down-sampling and network aggregation

**DOI:** 10.1038/s41598-019-50885-8

**Published:** 2019-10-08

**Authors:** Franziska Liesecke, Johan-Owen De Craene, Sébastien Besseau, Vincent Courdavault, Marc Clastre, Valentin Vergès, Nicolas Papon, Nathalie Giglioli-Guivarc’h, Gaëlle Glévarec, Olivier Pichon, Thomas Dugé de Bernonville

**Affiliations:** 1 EA2106 BBV, Université de Tours, Tours, 37200 France; 2 EA3142 GEIHP, Université d’Angers, Université Bretagne-Loire, Angers, 49100 France

**Keywords:** Data processing, Gene regulatory networks, Plant sciences, Regulatory networks

## Abstract

Large-scale gene co-expression networks are an effective methodology to analyze sets of co-expressed genes and discover new gene functions or associations. Distances between genes are estimated according to their expression profiles and are visualized in networks that may be further partitioned to reveal communities of co-expressed genes. Creating expression profiles is now eased by the large amounts of publicly available expression data (microarrays and RNA-seq). Although many distance calculation methods have been intensively compared and reviewed in the past, it is unclear how to proceed when many samples reflecting a wide range of different conditions are available. Should as many samples as possible be integrated into network construction or be partitioned into smaller sets of more related samples? Previous studies have indicated a saturation in network performances to capture known associations once a certain number of samples is included in distance calculations. Here, we examined the influence of sample size on co-expression network construction using microarray and RNA-seq expression data from three plant species. We tested different down-sampling methods and compared network performances in recovering known gene associations to networks obtained from full datasets. We further examined how aggregating networks may help increase this performance by testing six aggregation methods.

## Introduction

**Figure 1:**
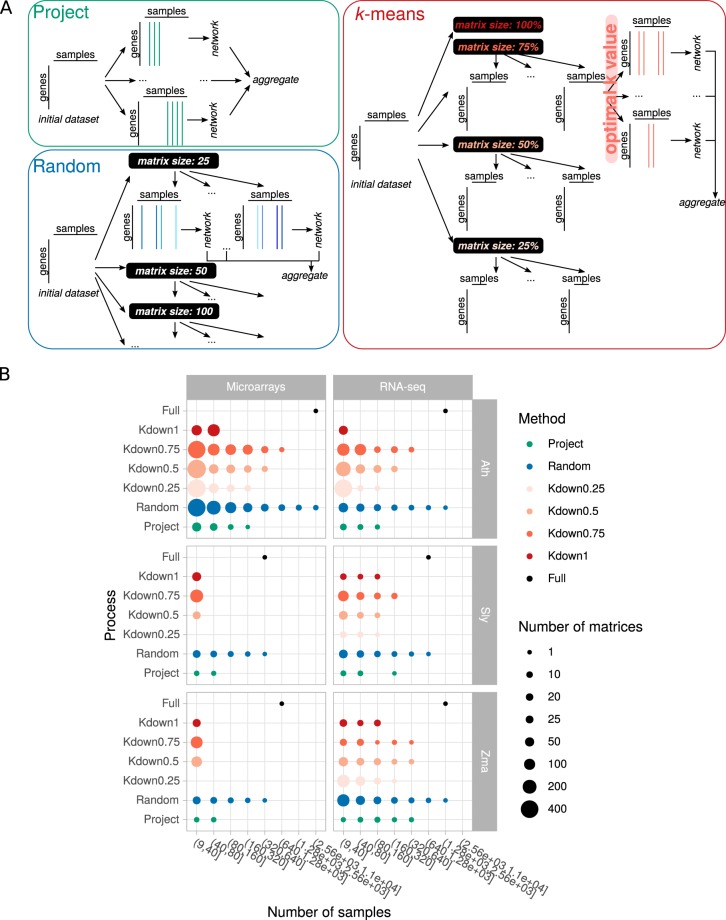
Down-sampling strategies. (**A**) Starting from a given number of samples retrieved from publicly available accessions, three methods were used to partition samples. Samples were clustered by project accession, by *k*-means or randomly. *K*-means clustering was performed on expression matrices containing 100, 75, 50 or 25% of the initial samples. For each set, samples were clustered with an optimal k value determined according to the elbow method (Suppl Fig. 1). Subsets were replicated several times. For the random down-sampling, several final sample sizes were tested with multiple replications. All subsets are then processed to generate networks which are further characterized. (**B**) Number of subsets obtained for each strategy. Kdown1, Kdown0.75, Kdown0.5 and Kdown0.25 respectively correspond to *k*-means clustering applied to 100, 75, 50 and 25% of the initial samples.

**Figure 2:**
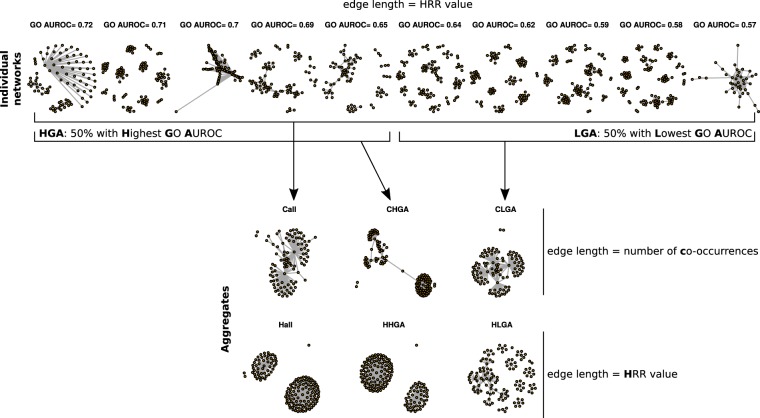
Network aggregation methods. The present examples focus on 10 individual networks with different GO AUROCs. Individual networks may be combined all together or only considering those with highest or the lowest GO AUROC (HGA or LGA). During aggregation, edges retain their HRR values in individual but their co-occurrence in several networks is also counted. Final aggregates contain either co-occurring edges (Call, CHGA and CLGA methods) or edges having the lowest HRR (more significant edges) (Hall, HHGA and HLGA methods).

Combining individual datasets (from independent studies, SRP/ERP numbers in RNA-seq or GSE in microarrays) is expected to increase biological situation range and help capture transient associations. Real gene pair associations will be found in the network only if their common expression is detected in the starting dataset. Including more datasets in co-expression analyses should therefore add biological situations where such co-expression may occur. Contrastingly, increasing the sample number in an expression dataset may also result in increased noise together with decreased capacity to detect transient associations, following the ‘garbage in garbage out’ principle. An open question remains about the number of expression datasets needed to build the most biologically relevant networks, *i*.*e*. capturing as many real associations as possible while keeping the number of false negatives low. If including more samples to construct a network improves its quality, it is still unclear how many datasets are sufficient to capture relevant gene associations. For model species with many available expression datasets (*e.g.* more than 1,000 samples), global networks can be constructed from one expression matrix combining every available sample but does it capture more efficiently biological associations than networks obtained from smaller or down-sampled datasets? How adding or removing samples alters the network composition is not clear. A pioneer study by Cosgrove *et al*.^1^ used an *Escherichia coli* microarray data compendium. They analyzed dependencies among samples and found that compendium subsets perform better than the full one in transcriptional regulatory network inference. The low efficiency of the global network was attributed to sample redundancies but could be circumvented by calculating an optimal effective number of samples. In their work, the full compendium (376 samples) could be down-sampled to 50% without decreasing network quality. Using a larger *E*. *coli* expression compendium (524 samples) as well as synthetic datasets (with up to 2,000 samples), Altay^2^ tested several information theory based inference methods as well as the sample size effect. In this case, both simulated and real datasets showed that *ca*. 100 samples were sufficient to capture transcriptional genome-wide regulations. Reports by Cosgrove *et al*. and Altay took advantage of the well known *E*. *coli* regulatory network to evaluate their co-expression networks. In another study^3^, co-expression networks were obtained after applying a Random Matrix Theory process to threshold similarity matrices calculated with Pearson Correlation Coefficients (PCC). The effects of both gene number and sample size were analyzed for 3 species: Human, Rice and Yeast. The authors have shown a high edge conservation between full and down-sampled networks. However, new edges appeared with smaller datasets (down to 25% of the initial size) while other edges were lost. This indicates that conserved associations between genes are easy to uncover while revealing more transient association typically depends on the nature of samples in the dataset. Conserved associations corresponded to functional associations, suggesting that genes added or removed in the down-sampled networks mostly were found in already densely connected modules and were weakly connected to others (*i*.*e*. they were not hub genes). Supervised down-sampling of a large dataset by finding the most appropriate dataset has been proposed to improve pathway reconstruction. Using a set of query genes, Hibbs *et al*.^4^ calculated correlations among this set on Single Vector Decomposition-transformed individual datasets of *Saccharomyces cerevisiae*. Each dataset was weighted according to its relevance, *i*.*e*. datasets maximizing PCC are given more weight. These weights are used next to calculate PCC of every gene with each query gene. This procedure is known as the SPELL algorithm (Serial Pattern of Expression Levels Locator). It has also been reported that creating subsets of related samples using a *k*-means approach improves feature detection^5^. However, it remains to be determined whether individual networks resulting from down-sampled partitioned datasets could be more efficient in capturing associations than a network calculated from all initial samples. Aggregated networks have also been shown to improve the recovery of biologically relevant associations^6,7^. The underlying idea is that conserved coexpression links between 2 genes over several datasets as termed by Lee *et al*.^6^ reinforce the existence of a true association between these 2 genes. A web-based tool named MEM was designed to merge co-expression lists obtained from individual datasets^8^. This multi-species microarray-based tool allows users to find the best coexpressed genes with an input query gene and manually excludes less relevant datasets considering the input. In MEM, gene ranks calculated with a query gene are aggregated over the selected datasets by using a binomial distribution hypothesis to attribute *p*-values to ranks and by taking the minimum value of all *p*-value. Ballouz *et al*.^9^ have constructed individual networks for different experiments and subsequently aggregated them, either taking all datasets or only the most significant (as indicated by Area Under the Receiving Operating Characteristic (AUROC) of GO terms). They have revealed a clear improvement over individual experiments, probably because it has the advantage of combining moderately significant or condition-specific relationships^9^. Using correlation networks constructed with PCC ranked with HRR (Highest Reciprocal Ranks)^10^, our aims are: (i) to establish the impact of sample size on the recovery of relevant associations at both global and targeted levels and (ii) get more insights on the way small individual networks (Fig. 1) may be aggregated to generate stronger networks (Fig. 2). Results presented here should help network construction hence optimize the recovery of biologically relevant associations using microarray or RNA-seq expression data. We provide in Table 1 a short description of the terminology and concepts used here.

**Table 1:** Terminology used in the present study.

Term	Definition
Sample	One column in the expression matrix corresponding to a specific experimental condition in which the expression of all transcript is measured
Subset	A down-sampled expression matrix
Down-sampling	Extracting part of all samples contained in a full expression matrix to create a subset.
Project	A group of samples from a same study
Network	A list of the strongest links (=edges = associations) connecting transcripts (nodes). In individual networks, a lower HRR value indicates a stronger link.
HRR	Highest Reciprocal Ranking: A ranking procedure of Pearson’s Correlation Coefficients
PLC	Pathway Level Coexpression: a subnetwork containing a set of guide genes and their associated genes.
Performance	Network and PLC performance is evaluated by their predictability regarding GO term annotations. It is quantitatively measured by the GO AUROC. It is quantitatively measured by the GO AUROC.
GO AUROC	An absolute measurement of network performance in associating gene pairs into known GO associations.
GG	Guide Gene: gene from a specific pathway used to extract other genes that are best co-expressed with it in a PLC.
Aggregate	A superposition of several networks. Overlapping edges may be combined according to their HRR value or their number of occurrences.

## Results

### Preparation of down-sampled datasets

Expression datasets were prepared for three plant species, *Arabidopsis thaliana* (Ath), *Solanum lycopersisum* (Sly) and *Zea mays* (Zma) (Table 2). We generated a total of 2,412 subsets using three different down-sampling methods (Fig. 1). Expression matrices partitioned according to their original project had fixed sample numbers and we had no control on this. Therefore, this method had the lowest representation in terms of subset numbers. For the *k*-means based approach, different *k* values were tested. For each *k* value, total within cluster sum of squares was calculated and plotted against *k* to estimate clustering quality. Elbows were observed for *k* values between 100 and 250 (Supplementary Fig. S1A). Optimal *k* values were selected for each dataset to minimize the total within cluster sum of squares while keeping *k* low (black dots in Supplementary Fig. S1A). Hence, between 100 and 250 subsets were generated for each initial expression matrix. However, it appeared that the size of resulting subsets was highly variable and did not allow a good comparison with the random sampling procedure especially for smaller subsets (Supplementary Fig. S1B). We therefore applied *k*-means based partitioning to randomly down sampled matrices at 25, 50 or 75% (Supplementary Fig. S1A,C). The objective was to determine whether random combinations of samples may be partitioned with *k*-means to generate networks. The investigation included a total of 1,462 expression matrices. We selected optimal *k* values for down-sampled matrices and efficiently generated subsets with lower sample sizes (Supplementary Fig. S1C). Concerning the random sampling based method, we tested different sizes ranging from 25 to 3,200 when possible. We down-sampled expression matrices with or without sample replacement but we did not find differences in these two methods (Supplementary Fig. S2). For the remaining of the evaluation, we proceeded with the method without replacement. Following the three above-described procedures, we generated 1,064 randomized, 1,231 *k*-means partitioned and 117 project partitioned datasets (Fig. 1).

**Table 2:** Dataset sizes.

	Microarrays	RNA-seq
*Arabidopsis thaliana*	22,178 × 10,138	33,602 × 1,676
*Solanum lycopersicum*	6,284 × 627	32,419 × 1,046
*Zea mays*	10,309 × 680	61,581 × 2,516

Number of genes × number of samples.

### Validation of the assessment

Our aim was to determine whether networks constructed from partial datasets are more predictive than those from complete datasets. Predictability was evaluated as the network performance in collecting edges that are labeled within GO (Gene Ontology) annotations. This performance corresponded to the mean of each GO term AUROC, using a neighbor voting algorithm with a 3-fold cross validation. We ensured that AUROC was not prone to overfitting as revealed by the homogeneity between folds of cross-validation, confirming that AUROC provides an accurate measure of network performance, even for the large number of edges considered here (Supplementary Fig. S3). AUROCs were also compared to *p*-values of GO term enrichment calculated with a hypergeometric law. Despite discrepancies for some terms, hypergeometric enrichment and AUROC expectedly were in good accordance suggesting an AUROC threshold of 0.6 to be considered a robust enrichment, a thereby a good network performance. To construct networks, we set edge numbers at defined values to compare the different down-sampling processes. We did not determine a specific significance threshold to construct the adjacency matrix. We rather explored network performances at different edge numbers (from 62,500 to 2 million) constituting gene pairs with the lowest HRR value (Supplementary Fig. S4). The largest size did not represent more than 2% of the total number of possible edges. Using this procedure, we did not take into account changes in HRR (Highest Reciprocal Rank) distribution that might arise from different sample sizes. In fact, we did not observe significant dependency between matrix size and the maximal HRR value up to 5 million edges (Spearman’s *rho* = −0.111). Network performance increased with network size for all datasets (Supplementary Fig. S4). However, this increase slowed down from 1 million edges for Sly and Zma. To compare the different down-sampling strategies, we next focused on 1 million edge sized networks as a compromise between network size and performance.

In Pathway Level Coexpressions (PLCs), global networks are queried with guide genes known to belong to the same global process to explore how they are transcriptionally related, as well as to find other genes with interesting features are co-expressed with them. The objective is to link additional genes encoding enzymes, transporters or transcription factors to specific processes. As described previously^10^, PLC quality not only relies on its GO AUROC but also on topological metrics, such as modularity and the ability to correctly associate genes into communities reflecting specific subprocesses. Representative networks are displayed in Supplementary Fig. S5. While GO AUROC will be a good indicator of PLC performance, the correct inclusion of a maximum of guide genes in accurate communities which can be quantified with the normalized Chi-squared metric is also important to ensure a good capture of the whole transcriptional landscape of the process.

### Performance of individual networks

**Figure 3:**
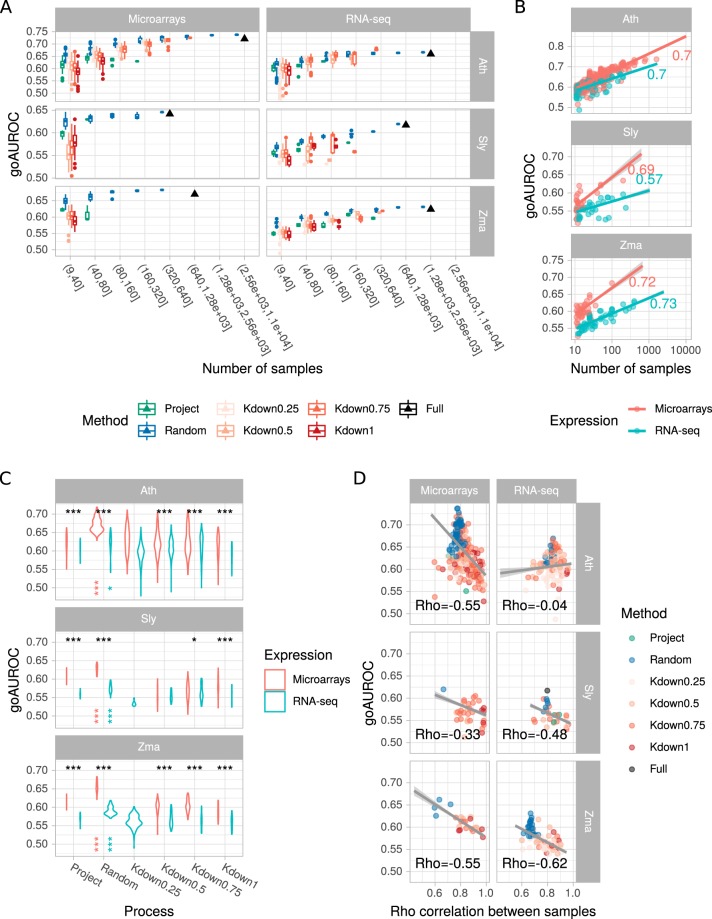
Performance of 1 million edge sized individual networks. (**A**) Influence of sample number in expression subsets on network performance. Boxplots summarize all networks obtained at a given size. Dots correspond to outliers. Black triangles correspond to result for full expression matrices. (**B**) Correlation between network performance and the number of samples in the subsets. Each dot represents one network. For convenience, only 10% of the networks are represented. Regression lines fitted on all networks with a linear model with 95% confidence interval are shown in grey. Values indicate Spearman’s *rho* calculated on all networks. (**C**) Comparison of network performance according to the down-sampling method and the expression data for subsets with less than 150 samples. Violin areas are proportional to the number of networks. Black asterisks denotate statistical differences in GO AUROC between microarray and RNA-seq. Colored vertical asterisks at the “Random” x-coordinate indicate a significantly different GO AUROC between the Random sampling procedure and the others. *p < 0.05; **p < 0.01; ***p < 0.001. (**D**) Relationship between network performance and correlation between samples in the subset. For convenience, only 10% of the networks are represented. Regression lines fitted on all networks with a linear model with 95% confidence interval are shown in grey. Values indicate Spearman’s *rho* calculated on all networks.

Networks were constructed from all possible subsets prepared as described above and their performance was measured according to their GO AUROC (Fig. 3A). At equivalent network size, network performance was globally higher when more samples were included to calculate distance between genes (global Pearson correlation coefficient 0.61) (Fig. 3B) and networks constructed from full data almost always displayed the highest GO AUROC (Fig. 3A). For all sample sizes considered, network performance of microarray datasets was higher than that of RNA-seq (Wilcoxon rank sum test *p*-value < 1e^−10^) (Fig. 3C). While GO AUROCs were high for genes analyzed by microarrays, these genes inherently represent less different processes than those measured by RNA-seq. It also suggests that genes pairs with a same GO term are more likely to be direct neighbors in microarray than in RNA-seq networks. A weaker predictability of RNA-seq derived networks was surprising. Because networks were obtained at the same number of edges, it indicated that many edges in these networks were not represented into the GO reference annotation. These edges could be considered either as false positive or true interactions not captured in the current GO reference annotation. It is possible that the more exhaustive view with RNA-seq encompasses gene associations which could be false positives or yet unaccounted true associations resulting in a decreasing in RNA-seq network predictability. It is also noteworthy that RNA-seq data are limited by the reference transcriptome used to quantify transcript abundance. For subsets with less than 40 samples, we found no clear trend for *k*-means partitioned datasets or subsets. For Ath and Zma, *k*-means applied to subsets containing 50% of the initial samples generated networks which performed slightly yet significantly better than networks constructed from full datasets partitioned with *k*-means (Student’*t* test, *p*-value < 0.05) (Fig. 3A). This indicates that a *k*-means partitioning can be applied on smaller datasets to generate subsets and networks without altering their performance. We found no statistical difference between project and *k*-means partitioning on network performance.

Randomly sampled subsets resulted in networks with a better performance than with the other down-sampling methods when considering subsets with less than 160 samples (Fig. 3A,C). This observation was less contrasted as more samples are used to construct the networks, probably because networks based on a larger number of samples performed better. Random down-sampling most probably included less related samples in comparison to projects or *k*-means. To verify this hypothesis, we calculated Spearman’s *rho* correlations between samples for each matrix and found that randomly selected samples were indeed less correlated ($rh{o}_{random}=0.75$ *vs* $rh{o}_{othermethods}=0.84$, Welch two sample *t*-test *p*-value < 2e^−16^). In fact, we observed an interesting trend in the relationship between sample relatedness and network performance. We found a weak negative correlation between sample correlation and GO AUROC of the resulting networks in all combinations but for Ath RNA-seq data (Fig. 3D). Although observed *rho* correlations between GO AUROC and sample relatedness was moderate (between −0.62 and −0.33), it seems likely that networks obtained from expression matrices with more heterogeneous samples may generate more predictive networks.

**Figure 4:**
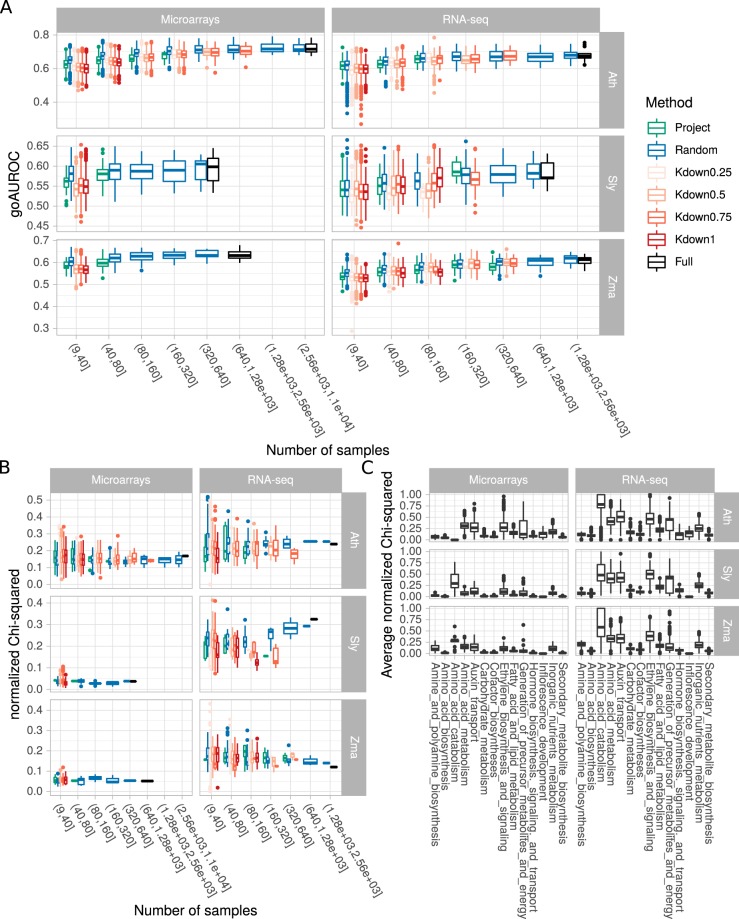
Performance of PLC constructed from individual networks. (**A**) Influence of sample number in expression subsets on the performance of 14 PLCs measured by GO AUROC. Boxplots summarize all networks obtained at a given size. Dots correspond to outliers. (**B**) influence of sample number in expression subsets on PLC ability to capture and correctly cluster guide genes measured by normalized Chi-squared. Results for only one PLC are shown (“Fatty acid and lipid metabolism”) because average normalized Chi-squared values strongly differed among pathways (**C**).

Concerning PLCs, down-sampling methods displayed no significant effect on their performance in capturing GO associations (Fig. 4A). However, performance was positively correlated to the subset sample size (all data combined, Spearman’s *rho* = 0.59). This was not the case for the normalized Chi-squared which measures how well guide genes are clustered within communities, taking into account the number of captured guide genes (for example with the guide genes from the “Fatty acid and Lipid metabolism”, Fig. 4B). This metric was highly variable among guide gene sets and among resulting PLCs (Fig. 4C). This observation suggests that some guide gene sets may not be totally well defined (*e*.*g*. in sub-process gene attribution), not correctly reconstructed or not integrated in the PLCs. It also indicates that highly variable qualities are observed in PLCs constructed from the down-sampled datasets and it was notably the case for subsets containing less than 100 samples (Fig. 4A,B). PLCs constructed from small datasets, *e*.*g*. for non model species with few available RNA-seq samples, should therefore be interpreted with caution. Evaluating individual networks indicated that the greater the sample number in the dataset the more predictive the network will be. However, we may hypothesize in turn that networks and especially PLCs generated from smaller subsets may capture transient but biologically relevant associations, only seen in specific experimental contexts and hidden in networks based on a large sample size. To test this hypothesis, we next tested whether aggregating individual networks resulted in an increase in GO AUROC and in PLC performance.

### Performance of aggregated networks

Individual networks constructed from down-sampled datasets were aggregated to determine whether GO capture can be improved (Fig. 2). Aggregates are expected to include either the most significant edges from each individual networks or the most frequent ones. The two approaches differ in that the most significant edges were not the most frequent. Taking Ath as an example, correlation between gene pair HRR value and number of occurrence was low (Spearman’s *rho* = −0.3 for microarrays and *rho* = −0.16 for RNA-seq). It is thus expected that aggregation mode will strongly impact final aggregate performance and properties. The main observations are described in the following paragraphs.

#### Aggregation methods result in different edge sizes

**Figure 5:**
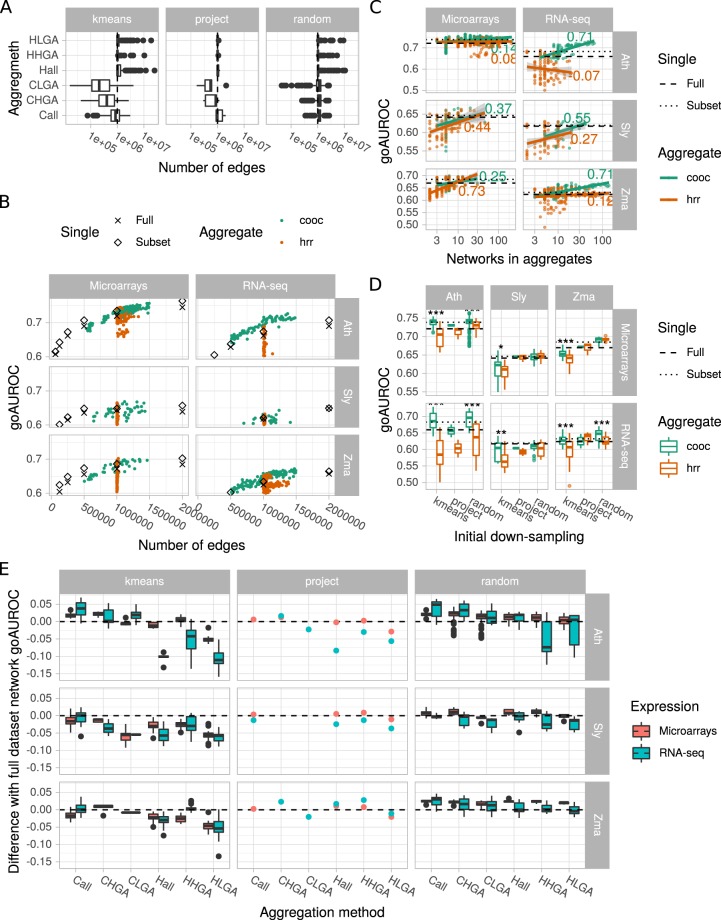
Network aggregate performance. (**A**) Number of edges in aggregates obtained with different aggregation methods. Dots correspond to outliers. (**B**) Aggregate performance as a function of edge number. GO AUROCs of individual networks (from full dataset or best subset) are represented by black symbols. Colored dots correspond to aggregates. (**C**) Correlation between aggregate performance and size. Dashed lines indicate the network GO AUROC of the full expression datasets and dotted lines the network GO AUROC of the best down-sampled dataset. (**D**) Effect of the down-sampling and aggregation methods on aggregate performance. Boxplots summarize all aggregates obtained for a given down-sampling and aggregation method. Dots correspond to outliers. Asteriks denote a statistical difference between co-occurrence and hrr aggregation methods (Wilcoxon rank sum test; *p < 0.05; **p < 0.01; ***p < 0.001). (**E**) Performance of aggregates relative to that of the network constructed from the full datasets. Positive values indicate a better performance in comparison to the full datasets. Boxplots summarize all aggregates obtained with a given aggregation method. Dots correspond to outliers.

In our aggregation methods, we tried to set aggregate size at 1 million edges to compare them more easily. However, final size was not as stable as expected (Fig. 5A). This was due to the fact that we used the HRR value or the co-occurrence number of the millionth best edge. To ensure an efficient aggregation, we retained all edges having this threshold and it sometimes included many more edges as observed in HRR-based aggregation modes (Hall, HHGA and HLGA). In addition, for the co-occurrence modes (Call, CHGA and CLGA), we retained edges that were found at least twice among all networks considered, which was not always possible, leading to smaller aggregates. For the remaining investigations, we only considered aggregates with more than 5 × 10^5^ and less than 1.5 × 10^6^ edges.

#### Some aggregates are more predictive than networks from full datasets or subsets

Aggregation modes were applied on networks constructed from subsets obtained with different down-sampling methods. We first questioned whether these aggregates may have a higher performance than networks constructed from full datasets or from subsets. In all cases, networks from subsets were slightly more predictive than networks from full datasets. We found that this was clearly the case for Ath and Zma RNA-seq and Sly and Zma microarrays (Fig. 5B). For the other species × expression combinations, the effect appeared more mitigated. Therefore, aggregates deserve deeper investigation.

#### Aggregate size is partially correlated to aggregate performance

Our aggregation procedures (see description in the Methods section) resulted in aggregates including different numbers of networks. In particular, for networks from randomly selected subsets, we investigated different aggregate sizes. We therefore examined how aggregate performance was correlated with the number of networks (Fig. 5C). We found a moderate correlation between these two parameters, depending on the species × expression combination. The strongest correlations were particularly observed for RNA-seq datasets and the co-occurrence aggregation method. As a consequence, it may be recommended to aggregate as many networks as possible to improve performance.

#### Co-occurrence based aggregation modes are more efficient

We next investigated how initial down-sampling methods and aggregation modes affected aggregation performance (Fig. 5D). Co-occurrence (cooc) based aggregates tend to be significantly more predictive than those based on HRR values (hrr), especially when aggregating networks constructed from *k*-means partitioned datasets (all data combined, +0.02 in GO AUROC for the co-occurrence method *vs* −0.016 in GO AUROC for the HRR method, Student’s *t* test, *p*-value < 2e^−16^). *K*-means partitioning was more efficient to create aggregates when initial datasets contained all or 75% of the initial samples (Supplementary Fig. S6A). Aggregates of networks from randomly selected subsets had a high performance in all combinations tested and may be probably used as a safe approach to generate high confidence co-expression networks. We found no real difference among initial subset sizes although aggregates of networks constructed with subsets containing 25 samples always gave good results (Supplementary Fig. S6B). Project-based aggregates appeared to have a lower GO AUROC than the other aggregates. As shown above, some aggregates clearly had a higher GO AUROC than that of single networks constructed from full datasets or subsets.

#### No strong difference are observed among all/HGA/LGA aggregation modes

During aggregation, we considered all networks, the 50% highest or the 50% lowest GO AUROCs. Aggregate performance was compared to that of networks constructed with the full datasets (Fig. 5E). Although these different approaches did not strongly affect aggregate performance, co-occurence based aggregates increased on average for GO AUROC- (+0.013) but not HRR-based aggregates (−0.008). CLGA and HLGA, integrating networks with the lowest GO AUROCs (**C**ooc-**L**owest **G**o **A**uroc and **H**rr-**L**owest **G**o **A**uroc), globally resulted in weaker GO AUROCs than Call or Hall integrating all networks (+0.005 *vs* +0.017 and −0.018 *vs* −0.002). CHGA and HHGA, which integrated networks with the highest GO AUROCs (**C**ooc-**H**ighest **G**o **A**uroc and **H**rr-**H**ighest **G**o **A**uroc) had performances very similar to that of the Call (all networks by cooc) or Hall (all networks by hrr) based aggregates. In aggregates of project-based networks, CHGA and HHGA gives good performances, but very similar to that of the full network.

#### Co-occurrence based aggregates have good metrics in PLCs

**Figure 6:**
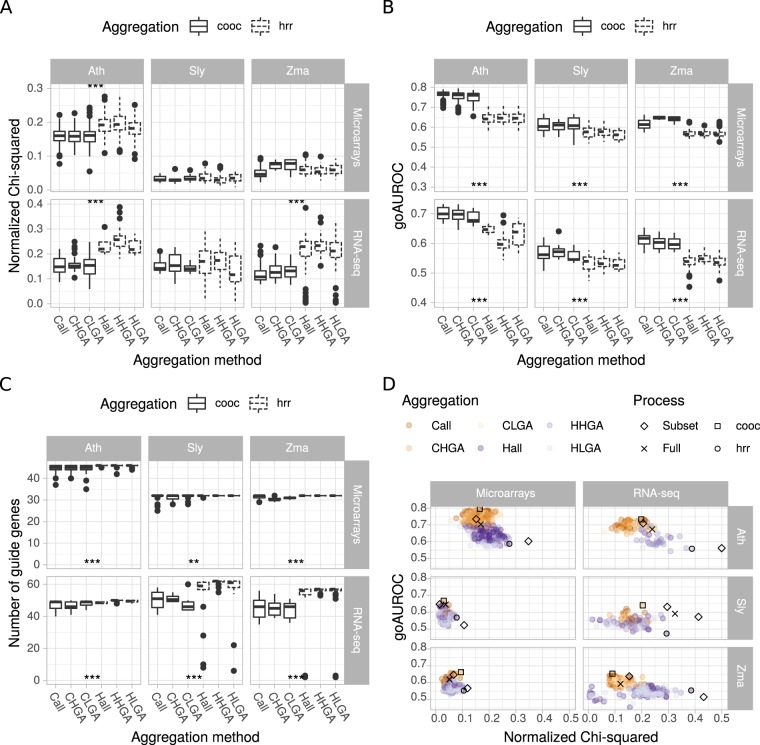
Performance of aggregated PLCs. Effects of the aggregation methods were evaluated on Normalized Chi-squared (**A**), GO AUROC (**B**) and the number of recovered guide genes (**C**). Asterisks denote statistical differences between cooc- (plain lines) and hrr- (dashed lines) based aggregation methods (Wilcoxon rank sum test; *p < 0.05; **p < 0.01; ***p < 0.001). Results for only one PLC are shown (“Fatty acid and lipid metabolism”) because average normalized Chi-squared values strongly differed among pathways (see Fig. 3C). (**D**) Correlation between GO capture (GO AUROC) and guide genes representation (normalized Chi-squared). All networks represented by black symbols were used in the jasmonic acid targeted PLC (Fig. 7). They correspond to individual networks (from full datasets or subsets) or to aggregates obtained either by co-occurrence (with the highest GO AUROC) or by hrr (with the highest normalized Chi-squared). Subsets represented by diamonds correpond to individual networks or aggregates with either the highest GO AUROC or the normalized Chi-squared.

We further evaluated the different network aggregation methods with respect to PLC performance and guide gene representation (Supplementary Fig. S7). The evaluation was made with 14 Reactome pathways but for simplicity purposes, we focused here only on the “Fatty Acid and Lipid Metabolism” pathway. As observed in our previous work^10^, HRR-based aggregates tend to have a higher normalized Chi-squared value than those obtained with the co-occurrence method, showing a diverging trend between GO capture performance and guide gene representation (Fig. 6A). This was not significantly the case for all PLCs but on average, higher normalized Chi-squared values were obtained for HRR-based aggregates (0.16 *vs* 0.13, Supplementary Fig. S7A). PLC GO AUROCs followed the trends observed for whole networks (Fig. 6B). This was also observed when all pathways were combined (Supplementary Fig. S7A). Aggregated networks with the co-occurrence method clearly displayed higher GO AUROCs than those obtained with the rank-based method. We found no statistical difference among aggregates created using all or only part of the networks (HGA or LGA) as well as among initial down-sampling methods. The number of guide genes finally represented in PLCs was significantly higher in HRR-based aggregates (Fig. 6C), in accordance with the apparent higher normalized Chi-squared values. This aggregation method may therefore be envisaged for PLC. Correlation between GO AUROC and normalized Chi-squared was globally low (ranging from −0.08 and 0.15) (Fig. 6D). This suggests that only some aggregates may have good performance in both metrics, although both parameters cannot be maximized in one single aggregate or network. Taken altogether, these results suggest that co-occurrence based aggregation of networks constructed from randomly selected subsets produce a network with high performance. To further illustrate all these observations and estimate the best construction methods, we constructed a targeted PLC focusing on the jasmonic acid biosynthesis and signaling pathway and visualized how the different down-sampling and aggregation methods affect PLC quality.

### Application to jasmonic acid (JA) biosynthesis in plants

To further demonstrate why aggregating networks is useful to study biological pathways, we extracted PLC from RNA-seq networks containing a million edges with genes involved in the well described biosynthesis of JA^11^. We performed this JA specific PLC on different network types: those constructed from full initial expression datasets, individual networks obtained from subsets or aggregated networks. For networks constructed from subsets and aggregates, we investigated two metrics: JA PLCs were constructed for networks or aggregates with the highest GO AUROC among all tested or with the highest normalized Chi-squared value calculated for the previous “Fatty acid and Lipid metabolism” PLC (Fig. 6D). Aggregates with the highest GO AUROC were constructed through edge co-occurrence while those with the highest normalized Chi-squared were obtained according to the lowest edge HRR (*i*.*e*., highest co-expression rank). Each million edge network or aggregate (a total of 30) was queried with guide genes (GG) obtained from the Plant Metabolic Network databases (26 for Ath, 44 for Sly and 33 for Zma) and included lipoxygenases (LOX), allene oxide synthases (AOS) and cyclases (AOC) among others (Supplementary Table 3). PLCs were thus constructed as previously but we set vertex number to *ca*. 30 to ensure a very targeted approach and consider only the strongest gene pairs. Gene pairs were either selected on their HRR value (for full dataset networks, individual subset networks and aggregates constructed according HRR value) or their co-occurrence (aggregates constructed according to the gene pair representation). Following this procedure, JA PLC never had an HRR > 30 or a co-occurrence number <3. This HRR value has been shown to be a stringent threshold^12^. Hence, all JA PLCs with 30 vertices are expected to be high confidence closely focused on JA metabolism and signaling.

**Figure 7:**
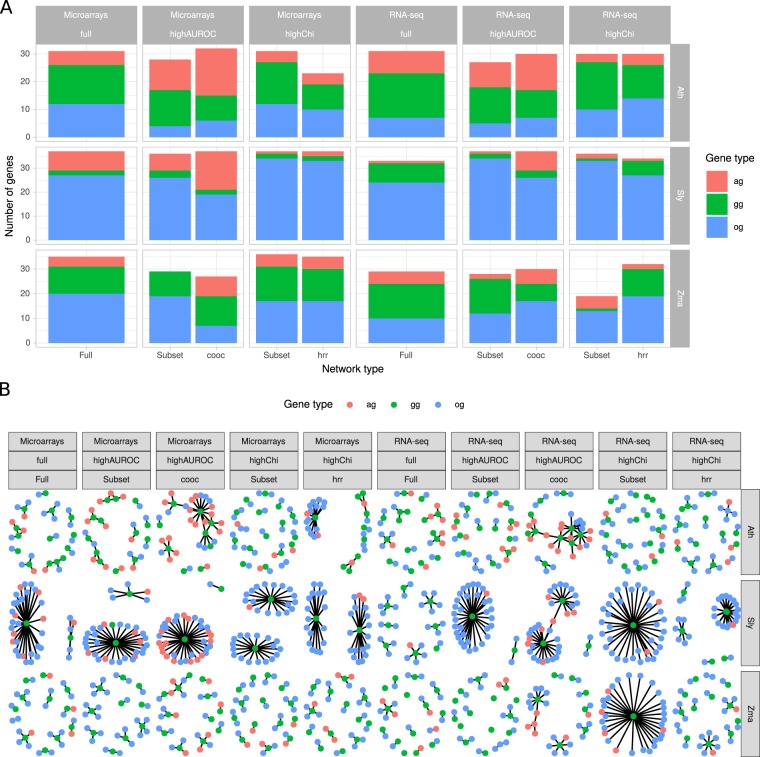
Application of co-expression networks, single or aggregated, to the Jasmonic Acid (JA) pathway. Networks with either the highest GO AUROC (highAUROC) or the highest normalized Chi-squared (highChi) were retained for each species × technology combination and among individual (No) or aggregated (cooc or hrr) networks. Networks from full initial expression matices (full) were also included. (**A**), number of associated genes (AG), guide genes (GG) and other genes (OG) in the JA PLC. (**B**), JA PLC visualization. Nodes correspond to genes and edges to gene pairs retained according to their weight or their HRR value.

Genes with evident relationships such as TIFY transcription factors, known modulators of the JA signaling pathway^13^, were classified as associated genes (AG) while those with no previously described relationship to this pathway were classified as other genes (OG) (Fig. 7A). PLC visualization clearly showed the strong influence of network construction procedure on PLC topology (Fig. 7B). Important features were the average node degree and modularity (Supplementary Fig. S8). Average node degree was globally higher in co-occurrence based aggregates than observed for full or HRR-based aggregates (Wilcoxon rank sum test, *p*-value < 0.05) while modularity was lower in co-occurrence based aggregates than in networks of full datasets (*p*-value = 0.03). This indicated more connections between nodes, and thus less isolated guide genes, in co-occurrence based aggregates with less hub-based structures although differences in log likelihood of fitting to a power law were not significant (Supplementary Fig. S8). While no difference was observed in the number of GG or OG among network construction procedures, co-occurrence based aggregates had a slight but significantly higher number of associated genes (Student’s *t* test, *p*-value = 0.023 *vs* individual subset networks, *p*-value = 0.014 *vs* HRR-based aggregates). Although not significant, co-occurrence based aggregates also tended to have more AG than full dataset networks (11.3 *vs* 5.1 respectively). These results suggest that co-occurrence based aggregates may be the most relevant PLC in this case. It is noteworthy that other aggregates or networks may also contain interesting and relevant associations. But in all cases, co-occurrence based aggregation generally included more information revealed by the higher number of associated genes.

Manual investigation of AG uniquely found in co-occurrence aggregates with the highest GO AUROC revealed interesting features (Supplementary Table 4). Firstly, several JA biosynthesis-related steps were found in these PLCs although not initially included as GG. This was the case for an allene oxide cyclase in Sly RNA-seq (Solyc02g085730.2) and a lipoxygenase in Zma RNA-seq (GRMZM2G015419). Next, several transcription factors were also identified, such as WKRY46 (AT2G46400) and ERF11 (AT1G28370) in Ath microarrays, an ERF-like (Solyc08g007820.1) in Sly microarrays, a jasmonate ZIM domain protein (Solyc12g009220.1) in Sly RNA-seq, a NAC-domain protein (GRMZM2G014653) in Zma microarrays or a bHLH domain protein (GRMZM2G301089) in Zma RNA-seq. All these domains are found in proteins known to be involved in plant defense signaling. Last but not least, we found interesting connections with plant specialized metabolisms as exemplified with a farnesoic acid carboxyl-O-methyltransferase (AT3G44860) in Ath RNA-seq, a cinnamyl alcohol dehydrogenase (AT4G37980) in Ath microarrays, an arogenate dehydrogenase (Solyc06g074530.1) in Sly microarrays and a dihydroflavonol-4-reductase (GRMZM2G013726) in Zma microarrays. These connections with the specialized metabolism probably highlight defense mechanisms activated upon stress relying on JA as a signaling hormone.

## Discussion

Using gene co-expression is an efficient approach to predict a gene function or find candidate genes involved in a given pathway (see for example^14–19^). In many cases, the authors generate their own data and infer co-expression from them. It is also possible to re-use published data to visualize transcriptional relationships in large expression compendia, such as those available on ATTED II^20^ and PlaNet^21^ databases. In any case, it is not clear how many samples should be included and/or how related they should be to calculate distances between genes. Specific datasets may highlight specific correlations while sharing many transcriptional relationships^22^. Whether a limited number of datasets is more appropriate than maximal datasets (containing all available samples) to capture gene transcriptional relationships remains to be determined.

Our results clearly showed that individual networks from down-sampled datasets (Fig. 3) rarely outperform networks obtained from the largest datasets, in accordance with previous studies^1–3,9^. For both microarray and RNA-seq, the more samples contained in the initial dataset, the higher the performance in capturing edges matching GO terms is expected to be. This was observed at both whole network and PLC levels (Figs 3B and 4A). Single networks from randomly down-sampled data subsets had a higher GO AUROC than single networks obtained from project or *k*-means partitioned data subsets. This trend was correlated with stronger similarities among samples in subsets obtained by these two latter methods (Fig. 3D). We also applied *k*-means to subsets variying in size in addition to full size datasets to generate smaller subsets. In Ath and Zma, groups of samples obtained by *k*-means applied to subsets generated slightly better performing networks (Fig. 3A,C). This could again be due to less similarities between samples.

Our results also show that microarray data resulted in higher GO AUROC at equivalent sample sizes (Fig. 3D). The higher AUROCs obtained with microarrays may reveal that single color arrays are well adapted to co-expression networks or that our annotation sets were more appropriated for genes effectively represented on each array. As gene models evolve with genome annotation refinement, RNA-seq, which is based on a mapping of reads on a reference transcriptome, allows to quantify more comprehensively gene expression^22^. For the three species investigated here, reference transcriptomes represented more genes than arrays. It is possible that the reference annotation sets used here reflected more array gene content than the more comprehensive RNA-seq based transcriptomes, suggesting that some associations in the RNA-seq network could be true positives but are considered as false positives because not found in the reference annotation sets.

Considering individual networks even with good overall performance may fail to identify transient associations between genes that may be biologically relevant in specific environmental conditions only (*e*.*g*. during a specific stress). Such transient associations should theoretically be highlighted more easily in networks constructed from smaller subsets, even if they had a weaker overall performance. In addition, we observed that smaller subsets may be better in grouping guide genes in PLC (Fig. 4B). We therefore considered combining individual networks and assessed the quality of the resulting aggregates. Aggregation has already been used in co-expression analysis^6,7^, however without extensive testing of aggregation methods. We tested here 6 network aggregation methods (Fig. 2).

Retaining edges co-occurring in several networks to construct the aggregate clearly improved performance in comparison to HRR based aggregation at whole network (Fig. 5A) and PLC levels (Fig. 6A). Some aggregates had a higher performance than that of full expression dataset networks at equivalent edge numbers. Aggregates of networks constructed from randomly selected subsets clearly had a higher performance than observed with other down-sampling methods at the whole network level (Fig. 5A). However, this was no longer the case at the PLC level (Fig. 6A). Concerning aggregation of networks constructed from *k*-means or project based partitions, their performance at the whole network level varied according to the species and expression technology.

In our previous work^10^, we showed that PLC quality could be monitored with the GO AUROC, normalized Chi-squared and modularity. This has previously been demonstrated using KEGG pathways on Arabidopsis. In the present article, we extended our set of validation pathway by considering the Reactome database. We found that it included more complete information especially for hormone signaling pathways and was more convenient to compare pathways between the three plant species investigated here. However, we found very low normalized Chi-squared values in contrast to the KEGG pathways. We impute this difference to the complexity of the Reactome pathways which sometimes included many different subpathways. Despite this difference, we similarly found a trade-off between PLC performance and the grouping of guide genes into communities (Fig. 6C). Our target example on the jasmonic acid in plants clearly show a better performance of aggregates retained according to their GO AUROC rather than their normalized Chi-squared (Fig. 7A,B). In this case, performance was also seen by a better capture of associated genes within the PLC. Such associated genes are really interesting candidates for a better understanding of biological pathways.

In the present article, we did not test other distance calculation methods. Whether our results are directly transposable to other more complex calculations (such as in supervised methods for regulatory network inference) remains to be determined. It is possible that the ranking procedure used here is tolerant to error and adapted to larger datasets. Mutual information based networks of *E*. *coli* expression data processed with different algorithms displayed similar trends during down-sampling^2^. Down-sampling had also a similar impact on network build with Spearman’s rank correlations^9^. Although more investigations will be required when using other distance measurements, it is likely that similar results will be observed with other methods.

## Conclusion

Taken altogether, our results suggest that co-expression networks using PCCs ranked with HRR clearly benefit from increasing sample size of the initial expression dataset. Small sized datasets (with less than 100 samples) had variable performance which was probably due to the samples they contained. We observed that differences between networks decreased when constructed from datasets with more than 100 samples. As a consequence, any combination of more than 100 samples may generate robust networks. When more than 500 samples are available (as it was the case in our 6 combinations), more biologically relevant networks can even be obtained by creating single networks after generating multiple subsets by randomly sampling the whole dataset and aggregating them according to co-occurring edges.

## Methods

### Dataset preparation

Microarray data were obtained from signal intensities in .CEL files downloaded from ArrayExpress in R^23,24^. Raw signals were quantile normalized with the RMA procedure in R^25^. Outlier arrays were detected by monitoring quartile distribution together with Kolmogorov-Smirnov testing against an empirical cumulative distribution curve^5^. RNA-seq data were obtained by downloading raw .fastq files from the EBI ENA. Reads were quasi-mapped with Salmon v.0.8.2^26^ on reference transcript assemblies obtained from Ensembl Plant to quantify transcript abundance. Assemblies were TAIR10 for *Arabidopsis thaliana*, SL2.50.31 for *Solanum lycopersicum* and AGPv3.31 for *Zea mays*. Matrix sizes are indicated in Table 2. All accessions are indicated in Supplementary Table 1.

### Dataset sub-sampling

Initial expression matrices were down-sampled in three different ways (Fig. 1A). Samples (columns in expression matrices) were combined according to their similarity (*k*-means), to their initial study (Project) or randomly. In the first method, *k*-means partitioning was performed in R using with the ‘h2o.kmeans’ function from ‘H2O’ package^27^. Columns were centered and scaled before applying the *k*-means algorithm. *K* values of 25, 50, 100, 150, 200 and 250 were tested for each dataset and the best *k* value was retained based on the total within sum of squares. *K*-means partitioned subsets were obtained from clusters containing more than 10 samples. *K*-means partitioning was also applied to randomly down-sampled datasets containing 75, 50 or 25% of the original samples. In this case, the retained samples were randomly selected without replacement. In the second method, datasets were partitioned by grouping arrays or runs from the same study/project (GSE for microarrays, SRP, ERP or DRP number for RNA-seq). Last but not least, we down-sampled datasets by randomly selecting a given number of samples. For each sample size, the random sampling without replacement was performed as many times as necessary to integrate all samples in one subset. For exploratory purposes, we also generated many subsets by randomly selecting samples with replacement (one sample can be found in one or more subsets, but cannot be found twice in one subset). In this case, the procedure with replacement produced many more subsets (5,116 in total vs 1,064 without replacement) because we ran it until at least 90% of the samples were represented at least once in a subset.

### Global network construction

Distances between transcript expression profiles were determined by calculating PCCs for each transcript pair. PCCs were next ranked so that for each gene, the rank value ranges from 0 (the gene itself) to N-1 (the total number of genes). For each gene pair, a Highest Reciprocal Rank (HRR) was given as the highest rank of the two genes, e.g. for a gene pair A and B, *HRR*(*A*, *B*) = *max*(*rank*(*cor*(*A*, *B*)), *rank*(*cor*(*B*, *A*)). Computations were done in parallel with an MPI program written in C^10^. In the HRR calculations, set of ties in PCC ranks were set as the minimal rank value of the set. Because threshold choice has been shown to strongly influence network topology^28^, we constructed and evaluated these global networks by retaining the best gene pairs from the whole list of HRR, ranging from 62,500 to 2 million edges. We opposed global networks (considering the n best gene pairs) to targeted networks also named Pathway Level Coexpressions (considering the *n* best gene pairs containing at least one guide gene, see below). When required, commands were run in parallel using GNU parallel^29^. R packages ‘ggplot2’^30^ and ‘reshape2’^31^ were massively used.

### Pathway Level Coexpression (PLC)

To test how genes from a given biological pathway were represented in global networks, we queried 1 million edge networks with different guide gene (GG) sets described in the Reactome database. The aim was to extract specific gene contexts from the whole networks. A total of 14 biological pathways were analyzed for each species. All gene pairs containing GG within the networks were retained to construct a pathway-targeted sub-network, a process known as Pathway Level Coexpression^32^. This approach captures associated genes (AG) that are strongly coexpressed with GG. In these sub-networks we also retained edges between AG that were found in the first million edges^10^.

### Global network and PLC performance evaluation

Global networks and PLC were evaluated for their ability to recover known or expected relationships between genes. Gene relationships obtained in a network were compared to gene associations described in Gene Ontology (GO) terms or the Reactome database^33^ (Supplementary Table 2). GO annotation files were downloaded from the Agrigo database v2.0^34^. Co-expression network performance was evaluated as its ability to capture gene pairs that are also found in the annotation network (obtained from the GO annotation). In other words, a gene pair in the co-expression network was considered as a true positive if both genes share similar GO terms. We applied a neighbor voting algorithm to measure how well connections in the co-expression network predict a given gene annotation. A three fold cross validation was performed and used to calculate an Area Under the Receiver Operating Characteristic (AUROC) for each GO term with the ‘EGAD’ R package^35^. Network performance was estimated using GO AUROCs which correspond to the average of all GO term AUROCs. GO AUROC scores of 0.5 and 1 respectively indicate random and perfect predictability. We also tested significant enrichment of networks with GO terms using a hypergeometric test with *p*-values adjusted by a Bonferroni correction. PLCs were evaluated in their ability to capture biologically relevant GO terms (GO AUROCs and significantly enriched GO terms) as well as to partition guide genes into their exact groups/pathway by measuring network modularity and a normalized Chi-squared metric as explained in Liesecke *et al*.^10^.

### Network topological metrics

Networks were constructed and analyzed with the R ‘igraph’ package^36^. Several topological metrics were calculated^37^. The average node degree corresponded to the average number of edges per node. Modularity was used to evaluate how modular communities detected were with a fast greedy algorithm. Transitivity measures the probability that adjacent nodes of a given node are connected. We also calculated a log likelihood of node degree to fit a power law distribution. All these metrics give an indication of how nodes are connected to others and may help to choose more biologically relevant networks.

### Network aggregation

Two aggregation methods were tested with different modes for each (Fig. 2). Starting from networks containing 1 million edges, gene pairs were aggregated either by their co-occurrence (“cooc”) in the other networks or according to their minimal HRR value (“hrr”). In the first case, the most frequent gene pairs will be found in the aggregated. In the second case, aggregates will contain the most significant edges. To construct aggregates containing a million edges, we sorted edges according to their increasing HRR value or decreasing number of occurrence and retrieved the HRR value or the number of occurrence of the millionth pair and set these values as aggregate thresholds. To avoid removing edges with similar HRR or co-occurrence numbers, we retained all edges with an HRR value below or equal to the threshold or with a co-occurrence number superior or equal to the threshold. In co-occurrence based aggregates, edges were retained only if they were found in at least two networks, *i*.*e*. their weight was equal to 2. As a consequence, some aggregates contained less than a million edges. Networks to aggregate were selected according to the initial down-sampling method. For project partitioned datasets, we considered all the networks constructed from each project. For the *k*-means partitioned datasets, we considered all networks constructed after applying one *k*-means clustering (at a set *k* value) to the full dataset or to one subset (down-sampled at 25, 50 or 75% of the full dataset). For random down-sampling, we aggregated different numbers of networks with replicate at each tested size when possible. Taking Sly microarray dataset as an example, we created 10 different aggregates of 10 networks from subsets containing 25 samples but only 4 different aggregates of 19 networks from subsets containing 50 samples. During aggregation, all or only half of the networks were combined. When only half of the networks were combined, we retained either the 50% Highest GO AUROC (HGA) or the 50% Lowest GO AUROC (LGA).

## Supplementary information

Supplementary Information

Supplementary Table 1

Supplementary Table 2

Supplementary Table 3

Supplementary Table 4

## Data Availability

All expression data and networks are freely available upon request.
