# Supplementary material for: Improved gene co-expression network quality through expression dataset down-sampling and network aggregation
Source: Sci Rep. 2019 Oct 8;9:14431. doi: 10.1038/s41598-019-50885-8 (PMC6783424; doi:10.1038/s41598-019-50885-8)

Improved gene co-expression network quality through expression dataset down-sampling and network aggregation

Supplementary Information

Franziska Liesecke[1,*], Johan-Owen de Craene[1], Sébastien Besseau[1], Vincent Courdavault[1], Marc Clastre[1], Valentin Vergès[1], Nicolas Papon[2], Nathalie Giglioli-Guivarc'h[1], Gaëlle Glévarec[1], Olivier Pichon[1], and Thomas Dugé de Bernonville[1,*]

[1] Université de Tours, EA2106 Biomolécules et Biotechnologies végétales, Tours, 37200, France

[2]EA3142 GEIHP, Université d'Angers, Université Bretagne-Loire, Angers, 49100, France

[*]Co-corresponding authors: franzi.liesecke@gmail.com, thomas.duge@univ-tours.fr

Supplemental Fig1: k-means down-sampling. Full or down-sampled (at 25%, 50% or 75%, multiple random sample combinations were tested, see Suppl Table 1) expression matrices were subjected to k-means partitioning for different k values. A, the total within cluster sum of squares is plotted against k values. Each dot represent one expression matrix (a total of 1,462 matrices). Black dots indicate k values that have been retained for further network construction and characterization. B, number and size of subsets in the full datasets partitioned with the k-means, project or random approaches. C, size of subsets obtained after applying k-means to randomly down-sampled full datasets.

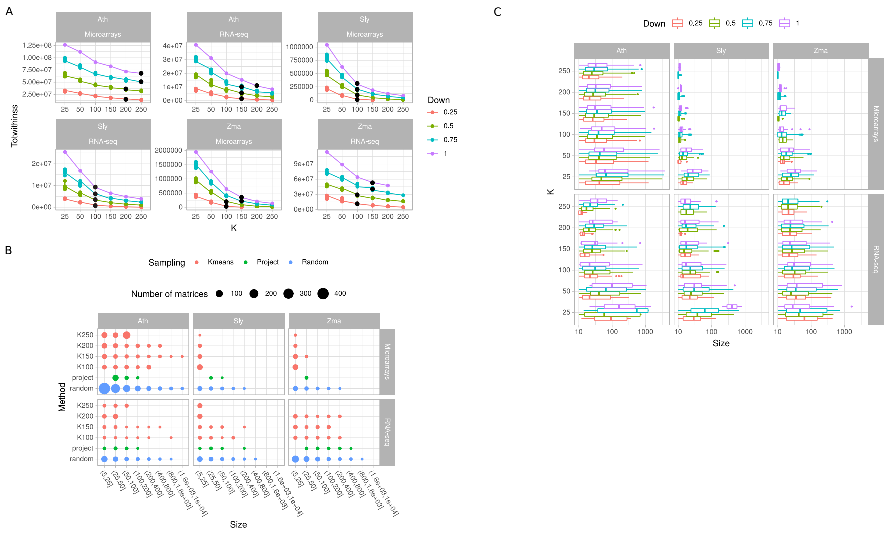

Supplemental Fig2: Effect of sample replacement on the performance of networks constructed from randomly sampled datasets.

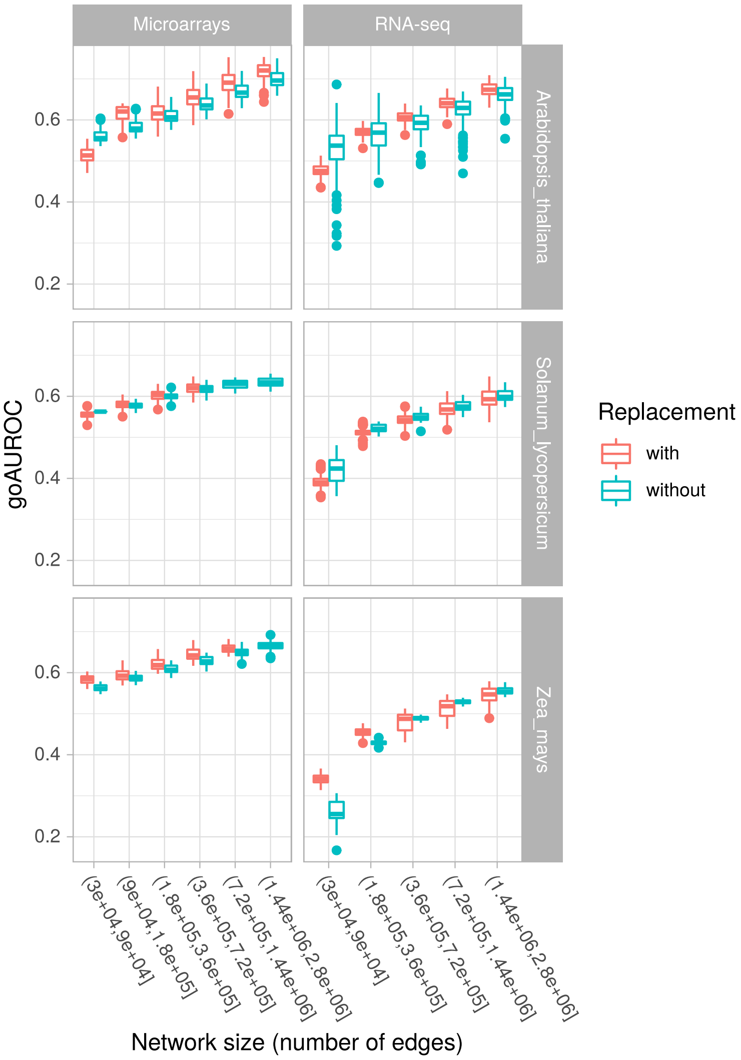

Supplemental Fig3: GO AUROC values in cross-validation. Data presented here came from the Solanum lycopersicum RNA-seq expression matrix. Different network sizes were tested ranging from 62,500 to 1 million edges. Left part: for each GO term found in the network, the 3 AUROC calculated within the cross-validation step are represented as boxplots. Points correspond to the adjusted p-value calculated from an hypergeometric test and their sizes are scaled to the gene number in each GO term in the network. Right part, comparison between GO AUROC and p-value of the hypergeometric tests. Each point corresponds to one GO term.

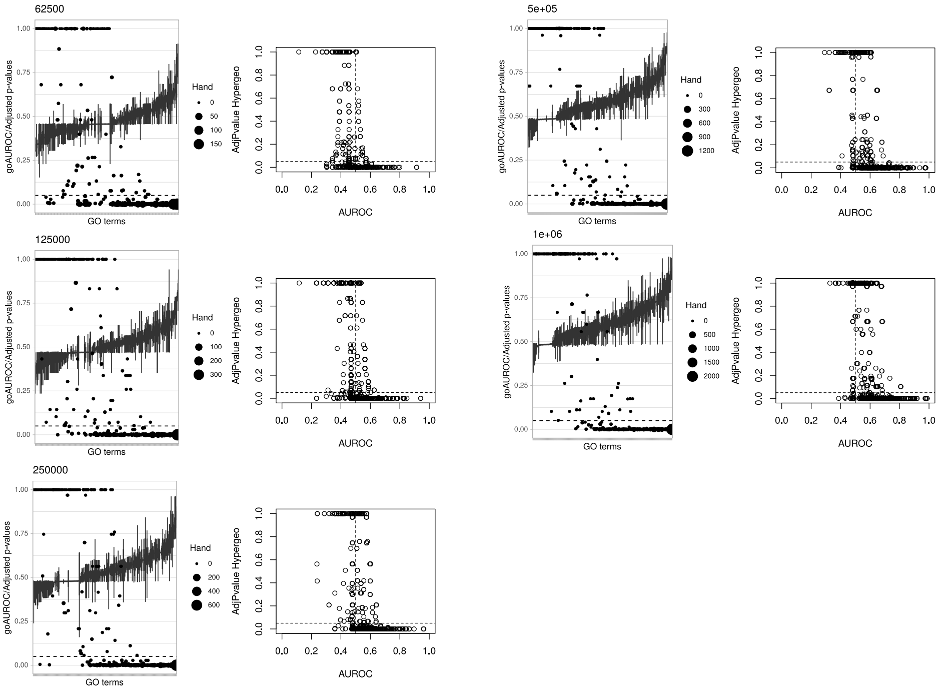

Supplemental Fig4: Effect of replacement in random sampling. Network performance is compared from subsets prepared by randomly selecting samples in the initial dataset, with or without replacement. Replacement implies than one sample may be used in different subsets.

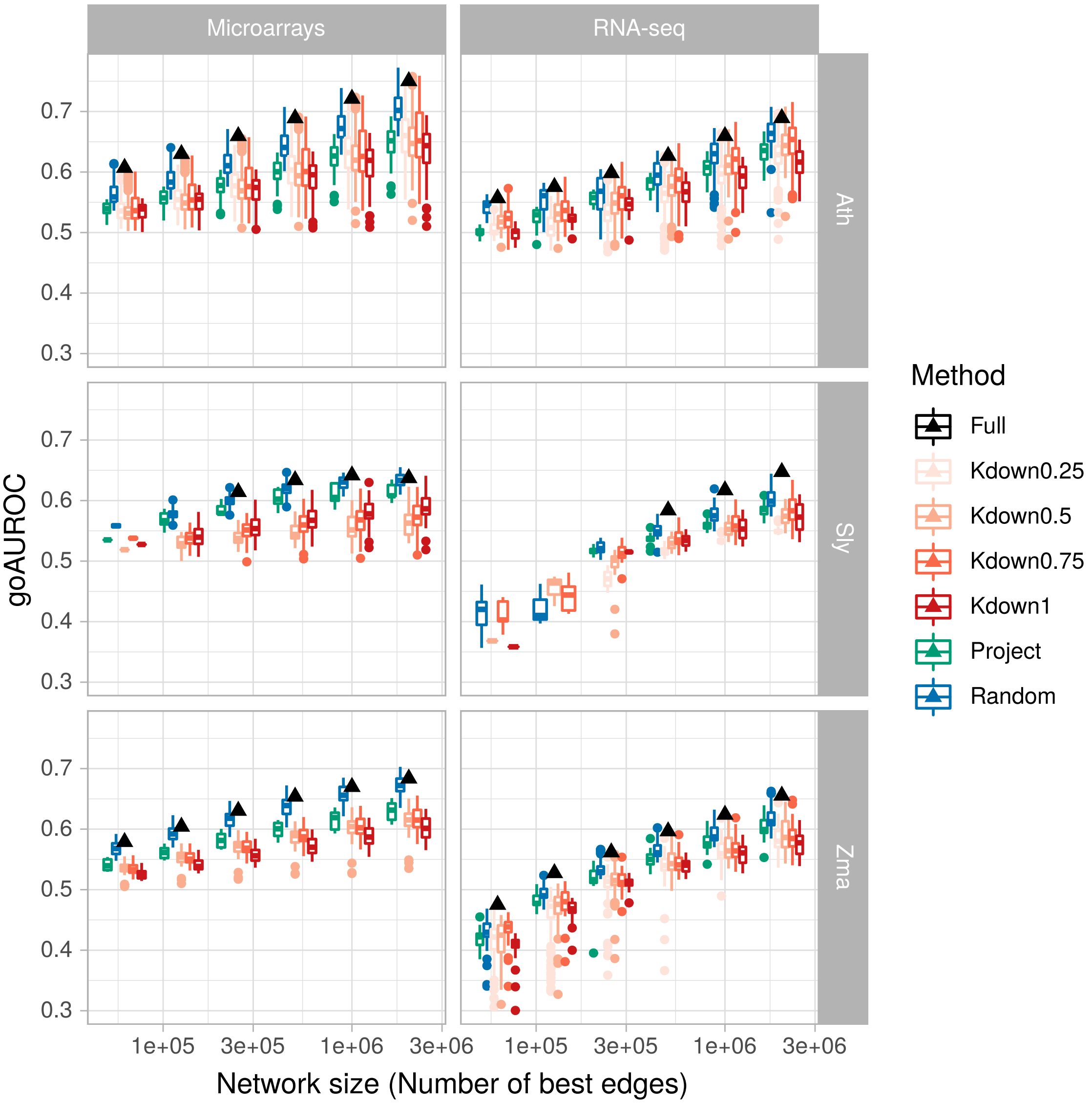

Supplemental Fig5: Estimation of PLC quality. The examples are drawn from different subsets of the Ath RNA-seq dataset. PLC quality may be evaluated by its transitivity (left), its ability to correctly associate query genes in communities (measured by a normalized Chi-squared test, center) or by its performance in capturing GO terms (right). Examples are given for low value (top) and high value (bottom) metrics.

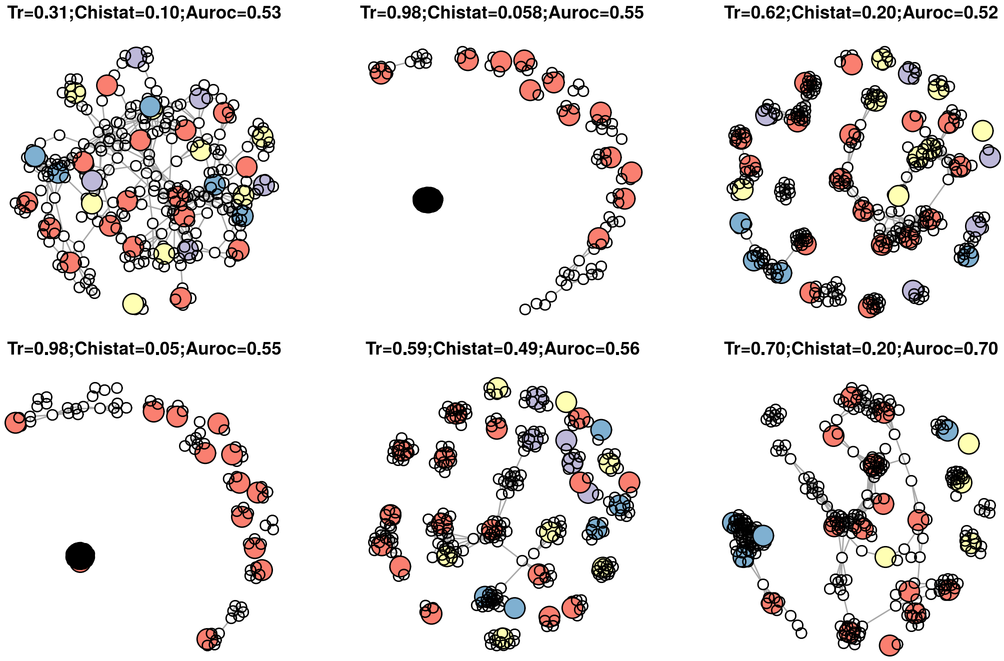

Supplemental Fig6: Effect of down-sampling methods on aggregate performance. Asteriks denote a statistical difference between co-occurrence and hrr aggregation methods (Wilcoxon rank sum test; *, p<0.05; **, p<0.01; ***; p<0.001). A, aggregates of networks obtained after applying k-means clustering full datasets or subsets containing 75, 50 or 25% of the initial samples. One aggregate contains all networks from one partition. B, aggregates of networks obtained after randomly selecting set of samples.

A

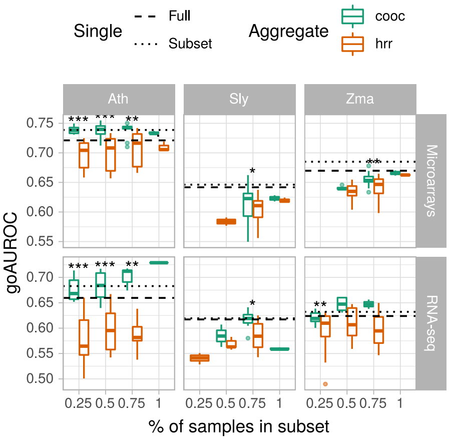

B

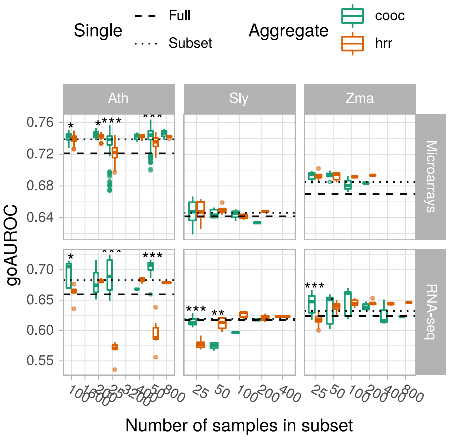

Supplemental Fig7: PLCs from aggregates. Performance of PLC was measured with normalized Chi-squared (A) and GO AUROC (B). Asteriks denote a statistical difference between co-occurrence and hrr aggregation methods (Wilcoxon rank sum test; *, p<0.05; **, p<0.01; ***; p<0.001).

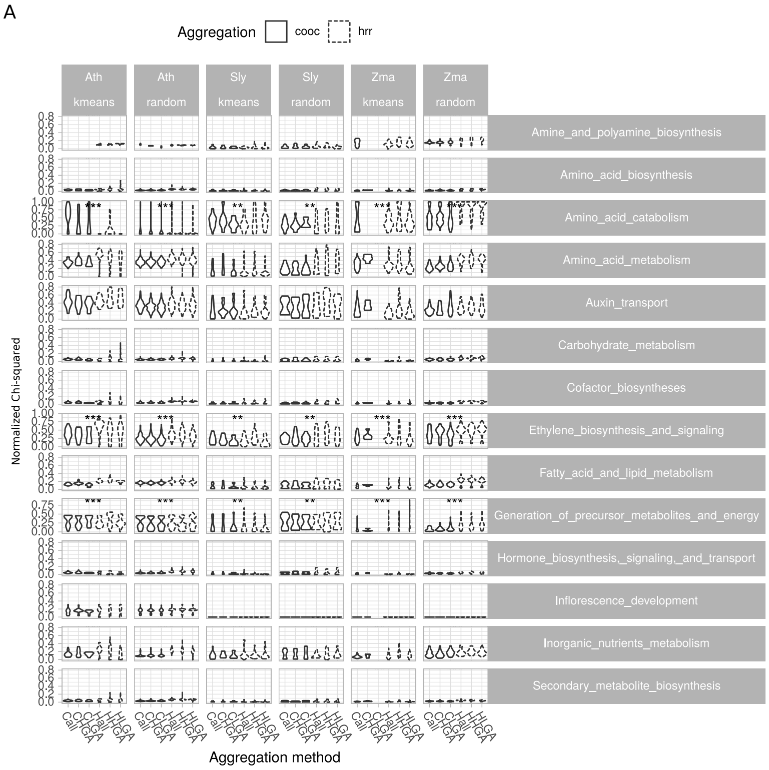

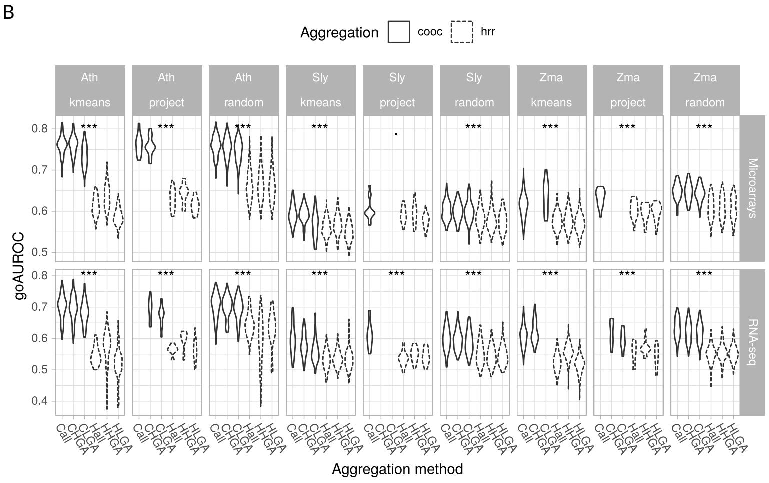

Supplemental Fig8: Topological metrics of Jasmonic Acid PLC. For each network or aggregate tested, PLC topology was evaluated by their modularity, transitivity, mean node degree and loglikelihood of fitting to a power law. PLCs are presented in Fig7C.

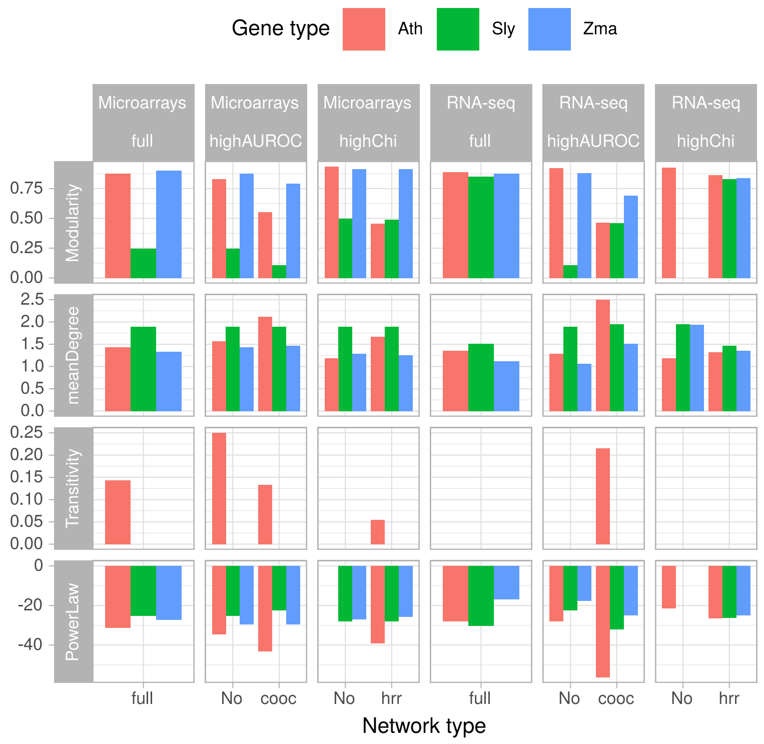

Supplement: Supplementary file 1 — Supplementary Information [file 41598_2019_50885_MOESM1_ESM.pdf]
